# Relationship Education and HIV Prevention for Young Male Couples Administered Online via Videoconference: Protocol for a National Randomized Controlled Trial of 2GETHER

**DOI:** 10.2196/15883

**Published:** 2020-01-27

**Authors:** Michael E Newcomb, Elissa L Sarno, Emily Bettin, James Carey, Jody D Ciolino, Ricky Hill, Christopher P Garcia, Kathryn Macapagal, Brian Mustanski, Gregory Swann, Sarah W Whitton

**Affiliations:** 1 Department of Medical Social Sciences Institute for Sexual and Gender Minority Health and Wellbeing Northwestern University Chicago, IL United States; 2 Department of Preventive Medicine Northwestern University Chicago, IL United States; 3 Department of Psychology University of Cincinnati Cincinnati, OH United States

**Keywords:** HIV/AIDS, relationship education, male couples, randomized controlled trial

## Abstract

**Background:**

Young men who have sex with men have a high HIV incidence, and a substantial proportion of incident infections occur in the context of main partnerships. However, romantic relationships also provide numerous benefits to individual health and wellbeing. 2GETHER is a relationship education and HIV prevention program for young male couples, and the 2GETHER USA randomized controlled trial (RCT) was launched to establish the efficacy of an online version of 2GETHER.

**Objective:**

The objective of 2GETHER is to optimize relationship functioning in young male couples as a method to improve communication about sexual risk behaviors and reduce HIV transmission. In the 2GETHER USA study, 2GETHER was adapted for online administration to couples across the United States via videoconferencing. The intervention in question aims to address the unique needs of couples from varied racial/ethnic backgrounds and geographic regions.

**Methods:**

This is a comparative effectiveness RCT of 2GETHER USA relative to existing public health practice (control). 2GETHER USA is a hybrid group- and individual-level intervention that delivers three weekly online group discussion sessions for skills delivery, followed by two individualized couple sessions that focus on skills implementation in each couple. The control condition differs by participant HIV status: (1) the Testing Together protocol for concordant HIV-negative couples; (2) medication adherence and risk reduction counseling for concordant HIV-positive couples; or (3) both protocols for serodiscordant couples. Follow-up assessments are delivered at 3-, 6-, 9-, and 12-months post-intervention in both conditions. Testing for rectal and urethral Chlamydia and Gonorrhea occurs at baseline and 12-month follow-up. The primary behavioral outcome is condomless anal sex with serodiscordant serious partners or any casual partners. The primary biomedical outcome is sexually transmitted infection incidence at a 12-month follow-up.

**Results:**

As of October 11, 2019, the trial has enrolled and randomized 140 dyads (Individual N=280). Enrollment will continue until we randomize 200 dyads (N=400). Assessment of intervention outcomes at 3-, 6-, 9-, and 12-months is ongoing.

**Conclusions:**

2GETHER is innovative in that it integrates relationship education and HIV prevention for optimizing the health and wellbeing of young male couples. The 2GETHER USA online adaptation has the potential to reach couples across the United States and reduce barriers to accessing health care services that are affirming of sexual minority identities for those who live in rural or under-resourced areas.

**Trial Registration:**

ClinicalTrials.gov NCT03284541; https://clinicaltrials.gov/ct2/show/NCT03284541

**International Registered Report Identifier (IRRID):**

DERR1-10.2196/15883

## Introduction

### Background

Young men who have sex with men (YMSM), including those in late adolescence and young adulthood, bear a disproportionate burden of the HIV epidemic in the United States [1]. However, there has not been a commensurate prevention response to curb the continued high incidence of new infections among these youth. Steady or main partnerships account for a large proportion of new HIV infections in men who have sex with men (MSM) (35-68%) [2,3], and this proportion may be much higher amongst YMSM (79-84%) [3]. Importantly, romantic relationships are much more than vectors of HIV risk for MSM; extant research on different-sex couples indicates that romantic relationships improve the health and wellbeing of individuals [4,5], and evidence suggests that these health promotive effects also apply to same-sex couples [6,7]. Thus, we developed the 2GETHER program to improve relationship functioning in young male couples and reduce the risk of HIV transmission [8]. The purpose of this manuscript is to describe the protocol for a randomized controlled trial (RCT) examining the efficacy of 2GETHER, delivered via videoconferencing technology to young male couples across the United States.

### HIV Transmission Risk in Young Male Couples

The large proportion of new HIV infections among YMSM that is attributable to main partnerships [2,3] is driven by multiple factors. First, YMSM are substantially less likely to use HIV preventive behaviors (eg, condoms, preexposure prophylaxis [PrEP]) when they enter into serious or main partnerships [3,9-11]. Further, nearly half of YMSM aged 13-24 who were HIV-positive in 2016 were not aware of their HIV status (29.1% of HIV-positive men who were 25-34 years old were unaware of their status) [1], so many HIV-positive YMSM may be entering into romantic relationships, reducing or eliminating their use of preventive behaviors, and then unknowingly exposing their partners to HIV.

Many male couples build “relationship agreements,” or arrangements that describe whether their relationship is monogamous or nonmonogamous and specify rules that delineate the conditions under which outside sexual partners are permissible [12,13]. Relationship agreements may be highly effective at minimizing the risk of HIV transmission or acquisition while maximizing satisfaction when the rules of such agreements are clear to and adhered to by both members of the dyad. Studies report varied estimates of the number of male couples who do not have an agreement in place [14]; however, several studies have found that a substantial proportion of those who do have an agreement disagree about their agreement rules [8,15,16], which may result in exposure to HIV (though we note that some studies have found less partner disagreement [17]). Further, breaks in relationship agreements (ie, noncompliance with agreement rules) are common in male couples (approximately 46% report breaks) [15]. When these breaks are not promptly disclosed to partners, couples risk damaging relationship trust, and if condomless or otherwise unprotected sex occurred, exposing one another to HIV. Key to building and maintaining relationship agreements is effective communication, and strategies are needed that provide YMSM with skills to establish and maintain effective agreements.

Binge-drinking and drug use have consistently been linked to engagement in HIV risk behaviors among MSM [18], and some evidence suggests that this link may be stronger among YMSM in relationships [19]. Further, substance use has been linked to a higher likelihood of breaking relationship agreement rules in male couples [20], as well as a higher likelihood of having condomless anal sex with extradyadic partners [21]. Finally, heavy alcohol and drug use are robust predictors of relationship discord, particularly when partners report discrepant patterns of use [22,23]. Thus, it may be particularly important to enroll young male couples who binge-drink or use illicit drugs in couples-based relationship education and HIV prevention efforts. However, we note that focusing exclusively on heavy substance-using samples may overlook the important risks to both sexual and relationship health of those who use substances but do so less frequently.

### Relationship Education and Couples-Based HIV Prevention

Existing approaches to couples-based HIV prevention have primarily focused on the provision of HIV testing and sexual risk reduction counseling in a couples format, particularly in Africa and other global settings [24]. Testing Together (formerly Couples HIV Testing and Counseling) is a Centers for Disease Control and Prevention–endorsed single-session HIV prevention strategy that is increasingly being used with HIV-negative YMSM in seroconcordant and serodiscordant (ie, one partner HIV-negative, one partner HIV-positive) relationships domestically [25]. This HIV testing strategy, which addresses some aspects of relationship functioning (eg, relationship agreements), has been adapted for remote administration via videoconferencing [26], and it has been enhanced to address substance use in male couples [27] and to include medication adherence counseling for HIV-positive individuals in serodiscordant couples [28]. However, given that it is a single session, Testing Together does not provide comprehensive relationship education content, which is key to establishing and maintaining safe and effective relationship agreements. Other couples-based HIV prevention programs that teach HIV risk reduction to both members of the couple simultaneously have been developed for heterosexual couples domestically and globally, with some providing more comprehensive relationship education skills training to enhance HIV prevention uptake [24,29,30]. However, very few such programs have been developed for male couples, and those that do exist have tended to focus on heavy substance-using couples [31].

There are several important gaps in couples-based HIV prevention for young male couples. Many YMSM, particularly those in serious relationships, are uninterested in programs that solely focus on HIV prevention, but YMSM report a strong interest in relationship education [32]. Thus, providing YMSM what they want (eg, relationship skills) while giving them needed HIV prevention skills, is a promising strategy for improving young male couples’ health and wellbeing. Further, most existing couples-based approaches do not adequately address secondary prevention among HIV-positive YMSM (ie, onward transmission of HIV from HIV-positive persons). Even those programs that do include HIV-positive individuals most often focus on reducing HIV transmission in serodiscordant couples, rather than the broader sexual health needs of HIV-positive persons, including those of seroconcordant HIV-positive couples. Further, YMSM have unique developmental needs (eg, lack of relationship experience, family stigma) that affect their ability to navigate sexual health in their relationships [33], and existing couples-based HIV prevention protocols do not address these issues.

Relationship education is a field that aims to promote long-term couple health by teaching skills to form and maintain healthy relationships, thus improving dyadic functioning in the present and preventing future discord [34]. Relationship education programs place a heavy emphasis on building effective communication and conflict resolution skills. These strategies’ effectiveness is supported by meta-analysis, which concluded that relationship education is effective in improving conflict-management skills and global relationship satisfaction [35]. Whitton and colleagues conducted some of the seminal work to adapt evidence-based relationship education programs for same-sex couples, and they have demonstrated acceptability to both female and male couples, as well as positive effects on couple communication, conflict resolution, and relationship quality [36,37].

The 2GETHER intervention’s unique contributions are that it uses evidence-based relationship education as a platform to deliver HIV prevention and sexual health promotion skills and that it has adapted this integrated relationship education and HIV prevention program to the unique developmental needs of YMSM [8]. Briefly, 2GETHER utilizes a hybrid group and individual format to teach various skills related to relationship and sexual health. The intervention demonstrated evidence of feasibility and high acceptability in a nonrandomized pilot trial with 57 young male couples in Chicago [8]. Further, the pilot trial showed evidence of preliminary efficacy, including significant posttest reductions in HIV transmission risk behaviors and improvements in HIV prevention motivation, mutual understanding of relationship agreement rules, and relationship investment. 2GETHER was the first program to integrate relationship education and HIV prevention for young male couples of any HIV status arrangement, including prevention content for concordant HIV-negative, concordant HIV-positive, and serodiscordant couples. Further, 2GETHER places an equal emphasis on relationship skills acquisition and sexual health, while existing programs have either emphasized HIV prevention or relationship education.

### Telehealth and Implications for Couples-Based HIV Prevention

The vast majority of health care services that are affirming of lesbian, gay, bisexual, transgender, and queer (LGBTQ) experiences are concentrated in the nation’s largest urban centers, creating wide disparities in access to services for individuals who live in suburban and rural locations. Indeed, rural MSM are substantially less likely to have received HIV/sexually transmitted infection (STI) testing and other preventive services and are more likely to report experiences of discrimination and bias due to sexual orientation [38]. Rural MSM in romantic relationships may be especially prone to stigma-based experiences because being partnered is a visible indicator of one’s sexual orientation. However, at the same time, a healthy and supportive couple relationship may help to buffer against the negative impact of such experiences [6]. Telehealth is an extensive field that focuses on enhancing health care, public health, health education, and service delivery using a variety of telecommunications technologies [39]. This strategy for service provision may help to reduce the gap in access to LGBTQ-affirming services between rural and urban MSM, but very few such telehealth programs exist for these populations.

As technology continuously advances, as does the media through which telehealth can be administered. Synchronous telehealth approaches are those in which interactions between the patient and provider occur in real-time, through telephone, videoconferencing, or real-time text interactions [40]. Asynchronous interventions, on the other hand, are those in which patient-provider interactions do not occur in real-time, and include Internet sites, Internet-based modules, or educational videos [41]. 2GETHER primarily utilizes a synchronous telehealth approach in which intervention content is delivered in real-time by live facilitators via videoconferencing technology to most closely mimic health care services delivered in vivo. 2GETHER uses asynchronous components (ie, narrated videos) to supplement live facilitation and minimize participant fatigue (described in more detail below).

Synchronous telehealth uses a variety of transmission technologies and devices, including telephones, computers, and personal communication devices [42]. Telephone-delivered treatments have been shown to be as effective as in-person treatments [41], but they are limited in their ability to capture nonverbal communication (eg, facial features, body positioning), which can be crucial in establishing rapport [43]. More recently, high-speed fiber-optic broadband networks have improved on these limitations and enhanced the capabilities of synchronous telehealth, bringing it closer to the experience of in-person treatment [44]. Advances in videoconferencing technology have also allowed for group video chat, so patients in different locations can participate in synchronous interventions in which they interact with one another, as well as with a health care provider [45]. Not only do group interventions allow for a larger patient-to-provider ratio, and thus are often used in settings where services are scarce [46], they also foster group unity and togetherness among participants [47]. Telehealth is also uniquely situated to overcome barriers commonly experienced with treating couples, in that: (1) coordination of multiple schedules is easier when couples can participate from home; (2) couples may be more open to sharing their experiences when they are not sharing the same physical space as facilitators and other couples; (3) there is a low likelihood that couples will run into facilitators or other participants in the real world; thus increasing willingness to participate; and (4) stigma associated with seeking treatment in brick and mortar settings at which they may be identified as a sexual minority is reduced [48].

The number of online HIV prevention programs designed for young and adult MSM is steadily increasing, but the vast majority of these interventions use an asynchronous approach that involves little to no live interaction with a facilitator [49,50]. Although asynchronous electronic health approaches are critical to improving the reach of LGBTQ-affirming and effective interventions, it is our belief that these automated approaches are not a replacement for the impactful live interactions with providers or facilitators that synchronous telehealth interventions provide. Concerning couple health, the ability to receive live coaching about relationship skills and sexual health allows couples to make changes in the moment, experiment with skill utilization, and observe the impact of these changes in vivo.

### Objectives and Aims

The goal of the current study is to conduct a comparative effectiveness randomized controlled trial to assess the efficacy of 2GETHER relative to existing public health practice in reducing HIV transmission risk and improving relationship functioning. We are recruiting a national sample of young male couples, who will complete intervention sessions remotely via videoconference. The purpose of this manuscript is to describe the protocol of the RCT.

## Methods

### Study Design

We are conducting a comparative effectiveness RCT to test the efficacy of 2GETHER relative to a control condition based on existing available public health practice. The control public health practice intervention will consist of a single session of either Testing Together [25], Medication Adherence Counseling [51], or both, depending on the HIV status of individuals in the dyad. We will randomize 200 dyads (individual N=400) to the 2GETHER intervention or public health practice, and we will examine primary and secondary outcomes at 12-months postintervention, with interim follow-up at 3-, 6-, and 9-months postintervention. The primary HIV risk behavioral outcome will be the occurrence of condomless anal sex acts with serodiscordant or unknown status partners (all casual sex partners will be considered unknown status), and we will account for the reduced risk of condomless anal sex in the context of PrEP use and undetectable viral load (eg, condomless sex while one has an undetectable viral load may be considered no risk). The primary biomedical HIV risk outcome will be STI incidence (ie, urethral/rectal Chlamydia and Gonorrhea). Secondary HIV-related outcomes will be indicators of engagement in the HIV continua of prevention and care, including HIV testing, PrEP use, and adherence for HIV-negative participants, and antiretroviral therapy adherence and self-reported viral suppression for HIV-positive participants. Other secondary outcomes include alcohol and drug use problems and indicators of relationship functioning. We will test for dose effects and decay in effects over time, and we will examine substance use problems and relationship functioning as mediators of change in HIV transmission risk. All primary outcomes will be measured at the individual level (not couple-level). This is advantageous because HIV risk may also occur with partners outside the relationship. Also, relationships may dissolve during the follow-up period, so measuring individual-level outcomes allows us to examine the effects of 2GETHER behaviors after relationship dissolution.

### Inclusion and Exclusion Criteria

Couples are eligible for this study based on the following inclusion criteria: (1) both members were assigned male at birth and currently identify as male; (2) both members are at least 18 years of age, and at least one member is aged 18-29; (3) both members consider one another to be their “main partner” (defined for participants as “…someone you feel committed to above anyone else. This would be someone you call your boyfriend, partner, or significant other”); (4) couple reports oral or anal sex with each another in the last three months; (5) at least one member reports having condomless anal sex with a known serodiscordant serious partner or with any casual sexual partner; (6) at least one member reports binge-drinking (ie, five or more drinks on a single occasion) or illicit drug use in the last 30 days; (7) both read and speak English at eighth-grade level or better; (8) both have access to the Internet; and (9) both agree to audio recording of intervention sessions.

Couples are ineligible if staff identify inconsistencies between information provided in the eligibility screener and baseline assessment (ie, a participant was faking eligibility or eligibility changed between screener and baseline), if issues arise that might hinder participation (eg, serious mental illness, intoxication), if both individuals are unable to be in the same place for the intervention sessions (ie, no long-distance couples), or if there is imminent risk for harm due to intimate partner violence. If either individual reports intimate partner violence (ie, their current partner has ever “hit, slapped, punched or physically hurt you” or “forced you to have sex when you didn’t want to”) at the baseline visit, study staff reach out via email to assess safety and provide resources. If participants report that they do not currently feel safe in their relationship, they are not eligible to participate in the study. These same procedures are followed if participants disclose intimate partner violence during their participation in the intervention sessions.

Concerning participant age (criterion 2), YMSM between the ages of 18-29 years old fall into the groups that currently have the highest HIV incidence [1], but we will allow one partner’s age to be 30 or older because age discordant partnerships are a risk factor for HIV acquisition among YMSM [52]. We require that there be some indication of HIV transmission risk (criterion 4) to increase the relevance of HIV risk reduction content. A past 30-day substance use criterion (criterion 5) will enroll couples for whom substance use is more likely to contribute to HIV-risk behavior and relationship conflict. Finally, we do not require a minimum relationship length, because our research has found that YMSM stop using condoms and other preventive behaviors when they consider their relationship to be “serious,” which often occurs very early in a new relationship (ie, less than three months) [9,53].

### Recruitment, Eligibility Screening, and Couple Confirmation

Participants are recruited using paid advertising on social media sites (eg, Facebook, Instagram), geospatial dating/hookup apps, and organic online engagement through social media posts (eg, Reddit, Twitter). Advertisements and posts direct the initially recruited participant (ie, “partner 1”) to a brief online eligibility survey, which includes an infographic illustrating study timeline and details. Eligibility surveys are administered via REDCap [54]. “Partner 1” is given the option to provide study staff with their partner’s contact information, so that we may send a confidential link to the eligibility survey for “partner 2.” If not provided, study staff contact “partner 1” to provide more information about the study and obtain the contact information of “partner 2.”

**Figure 1:**
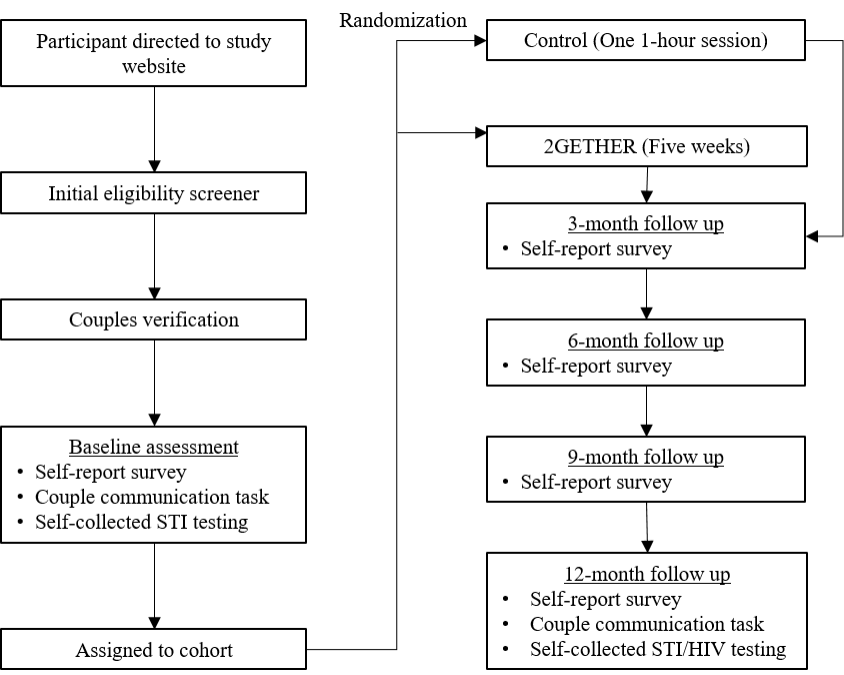
Flowchart of Study Timeline. STI: sexually transmitted infection.

Upon completion of eligibility screening of both partners, preliminarily eligible couples will complete a verification process. Study staff will perform phone call verification with each member of the dyad individually to confirm participant contact information and ask a series of questions to determine whether the couple is indeed two individuals in a romantic relationship. Couple verification includes asking questions about the participant’s partner (eg, “How old is [partner name]?”, “What is your partner’s address?”) and relationship history (eg, “How did you two meet?”, “Where was your first date?”). Once both calls are completed, the study staff will determine the couple’s eligibility to proceed in the study based on the response consistency of both members of the dyad. Individuals in eligible couples are then sent a link to the online informed consent and the baseline self-report survey and will be mailed materials and instructions for STI testing. After both members of the couple complete all components of the baseline assessment, couples will be scheduled for intervention sessions and randomized to one of the two intervention conditions. See Figure 1 for the flow of events for participants.

### Randomization to Treatment Arm

Couples will be scheduled into a group of 2-6 couples based on availability, and randomization occurs at the group level. Groups are assigned to either the active (2GETHER) or control (public health practice) condition using a covariate-adaptive randomization method [55,56], known as minimization. Minimization can control imbalance in baseline variables more efficiently than simple or restricted randomization and can manage a higher number of prognostic factors compared to stratification [57]. Minimization is uniquely suited to the current study design that schedules couples into groups, as it allows sequentially recruited clusters (eg, 4-12 individuals) to be treated as single units while balancing both individual- and couple-level prognostic variables [58]. For this trial, we used the range metric of imbalance in the minimization function and biasing probability of 0.80.

The allocation algorithm controls the imbalance on the following baseline factors: couple-level HIV-status (serodiscordant and seroconcordant positive), couple-level age discordance (one partner aged 30 or older), individual-level STI results (any positive result), and the total count of participants. We selected these prognostic factors because HIV risk (ie, outcome) may vary based on couples’ HIV status arrangement and may differ for age discordant partnerships; using these variables in the minimization algorithm will control against the chance of a large imbalance that may result in biased inferences. Positive STI result at baseline was added to the algorithm on October 15, 2018 (after 38 couples had been randomized) to control imbalance across conditions in this important variable that is linked to outcome.

Allocation concealment is assured in several ways. Participants are not eligible for randomization until all baseline components are complete, and at least two couples have committed to the same group session date(s). Dates are not, *a priori*, associated with study arm or intervention type. When a group is finalized, the data manager is immediately responsible for implementing the randomization algorithm and delivering the results to session facilitators. The randomization algorithm is stored in a secure, restricted, electronic location: only the data manager has access. The probability of assignment to the intervention yielding the least imbalance according to the algorithm (referred to as the biasing probability) is *P*=.80. Thus, there is always a random component to allocation to prevent against deterministic assigning and corresponding selection bias.

### Treatment Conditions

#### 2GETHER (Active)

##### Overview

2GETHER is a relationship education and HIV prevention program for young male couples that was developed based on formative mixed-methods research [19,32,59] and integration of components from the Strengthening Same-Sex Relationships program [37]. 2GETHER was initially designed as an in-person intervention, consisting of two group sessions focused on didactics, and two individualized couple sessions focused on skills implementation.

2GETHER teaches couples to use relationship education skills (ie, communication skills training, coping skills, problem-solving, acceptance) as a platform for optimizing their relationship functioning, improving their sexual health, and reducing HIV transmission risk. 2GETHER addresses HIV transmission risk in couples regardless of HIV status; couples learn to use behavioral and biomedical approaches to prevent both HIV acquisition (eg, HIV testing, condom use, PrEP) and transmission (eg, medication adherence to reduce viral load). Intervention content has been described in detail in manuscripts describing the nonrandomized pilot feasibility and acceptability trial [8] and a practical paper aimed at describing the implementation of relationship education for HIV prevention [60].

##### Online Adaptation and Pilot Trial of 2GETHER USA

To address the specific needs of young male couples outside of urban areas, we adapted 2GETHER for online administration (hereafter referred to as “2GETHER USA”) in two phases: (1) an initial content adaptation; and (2) a small pilot feasibility trial (N=10 dyads/20 individuals). During Phase 1, the study team reviewed the technical and usability merits of various videoconferencing platforms, followed by run-throughs of the group and individual sessions using the most promising platforms. We elected to use BlueJeans (BlueJeans Network, San Jose, California, United States) as our videoconferencing platform because it allowed for the highest degree of functionality (eg, hosting group chats, streaming video content live) and usability while minimizing technical issues (eg, strong connectivity, few interrupted sessions). Next, we completed several more rounds of internal content run-throughs and finalized study protocols for the Phase 2 small pilot feasibility trial. This involved finalizing protocols for national online recruitment strategies, remote baseline completion (including STI home testing and remote completion of couples communication tasks), and videoconference implementation of intervention sessions. We also made several alterations to address participant fatigue and enhance participant rapport, based on staff feedback and review of the telehealth literature [42]. First, we split the two group sessions into three sessions to minimize any loss of attention during remote sessions. Second, the group sessions involve a fair amount of didactic presentation, which is harder to follow for extended periods on videoconference. Thus, we prerecorded videos of the narrated didactic material (ie, PowerPoint presentations) and sent them to participants one week before group sessions (ie, three 20-minute self-paced modules per week). This facilitated briefer, more focused group sessions that emphasized discussion of intervention content and participant experiences that were already present in the original protocol. The structure and content of the individualized sessions, including live remote facilitation, were retained in 2GETHER USA. Finally, we developed specific training protocols to assist facilitators in speaking more clearly and conveying affect, which is more difficult through videoconference, in order to optimize rapport.

In Phase 2, we conducted a pilot trial of the adapted intervention with ten dyads (individual N=20). Participants were diverse in terms of race/ethnicity, HIV status, and geographic location. Participants completed a baseline assessment, consisting of three components: (1) online self-report questionnaire; (2) at-home testing for urethral and rectal Chlamydia and Gonorrhea; and (3) video-recording a couple communication task. They then completed group skill-building sessions, followed by individualized couple sessions for skill implementation. Upon completion of the intervention, participants completed a 2-week posttest and exit interview.

Regarding feasibility and acceptability, recruitment was rapid (ie, all participants recruited and enrolled January-February 2018), and couples were diverse in terms of demographics. All participants completed all intervention sessions and study components and reported few concerns with format or content. With regard to baseline assessment, some participants struggled to complete at-home STI testing and the video-recorded communication task promptly. We thus simplified instructions for these tasks and allowed couples to schedule appointments with staff to record the communication task remotely. We experienced occasional connectivity issues during videoconference sessions and difficulty coordinating participant schedules across time zones. We modified protocols to minimize these barriers (eg, simplifying technical instructions, scheduling by time zone).

##### 2GETHER USA Content Overview

The final 2GETHER USA program, after adaptation, piloting, and refinement based on participant and facilitator feedback, is comprised of five sessions. First, couples complete three videoconference group sessions aimed at skills building. Before each session, participants view three, 20-minute, self-paced video modules that address communication skills, coping with stress (both general and sexual minority-specific stress), relationship sexual satisfaction, and HIV transmission risk within the dyad and with outside partners. After viewing these modules, weekly one-hour videoconference group discussions led by two facilitators reinforce core concepts through structured conversations about how skills apply to couples’ relationships. We cannot guarantee that couples viewed the video modules, so each group session contains a review of core content. Participants are asked if they were able to watch the videos in order to guide the extent to which core content needs to be reinforced during group discussion. Videoconference groups are attended by 2-6 couples (both members of the couple must attend and be collocated) who can all see one another, with the screen enlarged on whomever is currently speaking, which helps to build community and facilitate group learning. If a couple does not show up to a group session, we proceed with the session (even if only one couple attends) and conduct a make-up session with the missed couple. In rare cases, couples may proceed with the intervention without having completed one or more group sessions, but we seek to incorporate missed content into remaining sessions.

Next, each couple completes two individualized couple sessions via videoconference with a program facilitator (with no other couples attending), aimed at skills implementation. The first individualized session focuses on communication skills coaching and problem-solving, in which couples discuss up to two areas of disagreement. Each partner communicates concerns, actively listens to their partner, and discusses problem-solving, with guidance and corrective feedback from the facilitator to facilitate effective use of these skills. The second individualized session, and the zenith of the intervention, focuses on sexual health. Utilizing effective communication skills, couples discuss sexual satisfaction within the dyad, their preferences for a monogamous or nonmonogamous relationship agreement, and biomedical and behavioral HIV prevention strategies. HIV-negative and unknown status participants receive HIV testing during this session, while HIV-positive participants and HIV-negative participants on PrEP receive medication adherence counseling, based on the Life-Steps protocol [51]. If participants test preliminary HIV positive, we provide participants with resources for confirmatory testing and linkage to care in their area of residence. At the end of the sessions, couples draft a detailed relationship agreement, which includes specific rules about monogamy or nonmonogamy and HIV prevention practices. After establishing an agreement, the couple discusses strategies for maintaining or altering the agreement in the future, as well as how they will handle agreement breaks if they occur.

#### Existing Public Health Practice (Control)

The public health practice intervention that couples in the control condition receive depends on the HIV-status of the partners: HIV-negative/unknown status participants receive a single-session of Testing Together [25], HIV-positive participants receive a single session of Medication Adherence and Risk Reduction Counseling [51], and serodiscordant couples receive both protocols in a single session. Testing Together, previously known as Couples HIV Testing and Counseling, is a public health strategy designed for two or more persons who are in, or planning to be in, a sexual relationship who receive HIV testing services together (including their HIV test results). Testing Together facilitates communication and disclosure of HIV status between the two partners, while also supporting linkage to HIV medical care, PrEP, and other appropriate services. Testing Together creates an opportunity for couples to discuss and prepare a risk-reduction plan based on the HIV status of both partners. Couples in which at least one member is HIV-positive receive Medication Adherence and Risk Reduction Counseling, which was developed based on Safren and colleagues’ Life-Steps protocol [51]. Based on cognitive-behavioral therapy principles, this session focuses on identifying motivations for and barriers to antiretroviral adherence, as well as making a plan for optimizing medication adherence and reducing HIV transmission risk. For serodiscordant couples who receive both Testing Together and Medication Adherence Counseling, the protocol emphasizes engaging each partner as an equal participant in both interventions, with the ultimate goal of reducing transmission risk within the dyad and with outside partners.

### Facilitator Training, Fidelity, and Supervision

All intervention facilitators hold a bachelor’s degree at minimum and have direct experience working in research or social service settings with young adults or MSM. Instead of emphasizing advanced education and training, our hiring practices prioritize community-based and direct service experience, including HIV testing and counseling, health education, teaching, counseling, research administration, and program coordination. Using bachelors-level facilitators (instead of mental health professionals) with relevant community-based experience means that the program will be easier to implement in community settings, which is an important consideration when designing interventions that contain group and individual session components. Given that all public health practice control content is also presented in the 2GETHER USA active condition, this study utilizes the same facilitators for the active and control conditions.

Each facilitator completed an intensive eight-week training protocol, which included Communication Skills Coaching, HIV Test Specimen Collection and Interpretation, HIV Risk Reduction, Testing Together, and session-specific intervention content. As part of the training, all facilitators completed mock session run-throughs with the Principal Investigator, Coinvestigator/Supervision Lead, and Project Coordinator for feedback. To reinforce facilitator skill-building, facilitators completed mock sessions with patient simulators who were given a case description (ie, individual characteristics, relationship history, relationship dynamics, and session-specific scripts). Patient simulation allowed facilitators to experience “real-life” sessions, as well as how to handle potentially negative or hostile situations, deliver HIV-positive test results, and guide and direct effective communication practice among dyads.

Facilitators will receive weekly supervision on their audio-recorded individual 2GETHER USA couples’ sessions. Supervision is primarily provided by one of three doctoral-level clinical psychologists and a masters-level HIV test counselor in a group setting. During group supervision, relevant segments of audio are played to illustrate both areas for improvement and ways facilitators skillfully handled difficult situations. As facilitators master the 2GETHER USA content, they are given opportunities to provide mentored peer supervision. Supervision for the public health practice control condition is conducted separately using an analogous format, led by a masters-level HIV testing counselor. Given that this trial uses the same facilitators across conditions, supervision aims to minimize drift in content across conditions by identifying moments when facilitators break condition fidelity.

To ensure fidelity to the intervention manuals, and thereby the essential components and content of the intervention, facilitators audio-record all 2GETHER USA and public health practice sessions. A total of 20% of sessions (both group and individualized couple sessions) are randomly selected for review by an independent assessor to validate appropriate content delivery. All staff members trained in intervention delivery will assist with fidelity monitoring. Facilitators are eligible to conduct fidelity assessments only for those couples with whom they did not work in either group or individual sessions to minimize bias. Fidelity monitoring assessors will complete a dichotomous checklist indicating whether or not the central components of each intervention session were completed and delivered effectively by facilitators. They will also rate facilitator time management, completion of collaborative activities, addressing participant concerns and questions, stimulating conversations, familiarity with session content and materials, and ability to develop a rapport with participants.

### Study Assessments

After participants complete couple verification, each individual in the dyad is sent materials to complete their baseline assessment, which consists of a self-report survey hosted on REDCap, a video-recorded couple communication task, and self-collected STI testing for urethral and rectal Chlamydia and Gonorrhea. The “baseline kit” contains detailed instructions for completing each component of the baseline. Based on prior work conducted in our group [61], we provide a guide for self-collection of STI samples and instructions for mailing the materials to the lab. STI testing results are delivered to each participant individually via phone, including referrals for treatment in the participant’s area of residence. Participants complete self-reported questionnaires at all follow-up points (ie, 3-, 6-, 9-, and 12-months postintervention), and they complete the couple communication task and self-collected STI and HIV testing at the 12-month follow-up. If a couple breaks up during the follow-up period, each individual still completes follow-up surveys and STI testing. If individuals then enter into new relationships, we gather information on their current relationship functioning in order to assess whether skills generalize to future relationships. Participants are compensated US $50 for completing each assessment time point, for a total of up to US $250 for each member of the dyad. See Figure 1 for the flow of events for participants and Table 1 for a list of primary and secondary outcomes by assessment timepoint.

**Table 1:** Primary and Secondary Outcomes and Assessment Schedule

Outcome type, construct		Measure/Operationalization	Measurement schedule				
			Baseline	3 months	6 months	9 months	12 months
**Primary**							
	HIV risk behavior	Condomless anal sex with a serodiscordant main partner or any casual partner [62]	✓	✓	✓	✓	✓
	STI^a^ incidence	Urethral and rectal Chlamydia and Gonorrhea: Aptima Combo 2 GC/CT nucleic acid amplification test [63]	✓				✓
**Secondary: dyadic HIV risk**							
	Relationship agreements	Partner concordance in (non)-monogamy agreement type and rules	✓	✓	✓	✓	✓
	Agreement breaks	Past 3-month breaks in (non)-monogamy agreement rules	✓	✓	✓	✓	✓
**Secondary: HIV prevention and care continua**							
	HIV/STI testing	Assessing past 3-month HIV and STI testing history	✓	✓	✓	✓	✓
	PrEP^b^ use and adherence	Current & past 3-month PrEP use; adherence over 7-, 30-, and 90-days [64-66]	✓	✓	✓	✓	✓
	ART^c^ adherence and viral suppression	Adherence over 7-, 30-, and 90-days; self-reported viral load (detectable/ undetectable) [65]	✓	✓	✓	✓	✓
**Secondary: relationship functioning**							
	Relationship satisfaction	Couples Satisfaction Index: 4-items [67]	✓	✓	✓	✓	✓
	Communication (self-report)	Communication Skills Test: positive and negative scales adapted [68]	✓	✓	✓	✓	✓
	Communication (objective)	10-minute recorded communication task [69-71], coded with Interactional Dimensions Coding System [72]	✓				✓
**Secondary: substance use**							
	Alcohol problems	Alcohol Use Disorders Identification Test [73]	✓	✓	✓	✓	✓
	Marijuana problems	Cannabis Use Disorders Identification Test-Revised [74]	✓	✓	✓	✓	✓
	Other drug use	Past 3-month use of prescription and illicit drugs [75,76]	✓	✓	✓	✓	✓

^a^STI: sexually transmitted infection

^b^PrEP: preexposure prophylaxis

^c^ART: antiretroviral therapy

### Analytic Plan

Chi-square tests and analysis of variance will be used to test for randomization imbalances on demographic factors, primary outcomes, and prognostic variables (ie, couple-level HIV-status, age discordance, and individual-level STI results) at baseline among the two treatment conditions. Observed imbalances will be adjusted for using baseline data in all subsequent analyses of treatment effects.

The primary biological outcome, change in STI prevalence rates between baseline and 12-month follow-up, will be examined using a Cochran-Mantel-Haenszel test of two independent binomial proportions. This test will allow for stratification while testing for significant associations between two binary variables. The primary behavioral outcome, condomless anal sex with casual partners or with serodiscordant main partners, and secondary outcomes will be assessed using multilevel growth modeling to adjust for the nested nature of our data. Initial power analyses to determine the sample size for 2GETHER was conducted based on individual-level outcomes so that partnerships breaking up throughout the study would have a limited effect on power, and power analyses assumed an approximate 20% attrition at 12-months. Latent growth curve factors will be formed for each outcome using data from the four follow up surveys (3-, 6-, 9-, and 12-month). Models will include the latent intercept and slope formed at the individual level for each outcome. The 2GETHER treatment condition will be entered as a dyad-level predictor of the latent intercept and slope. Significant treatment differences on the intercept will indicate differences in the outcome at the 3-month follow-up. By changing the referent time point for the intercept, we will also test for differences at 6-, 9-, and 12-months. Significant treatment differences on the slope term will indicate different trajectories of change for that outcome among the two study conditions.

For outcomes where significant differences have been identified based on treatment condition, relationship functioning, and substance use problems will be explored as potential mediating factors within the multilevel growth modeling framework described above. Variables will be identified as suitable mediators if, like the outcome, the treatment effect is related to change in the potential mediator. Mediated pathways will be identified using a parallel process approach where the treatment effect predicts change in both the mediator, which will be modeled as a lagged effect to maintain the temporal order necessary for mediation, and the outcome [77]. The indirect effect of the treatment on the outcome through the lagged mediator will be calculated using a percentile bootstrap test. To address partners who break up in analyses where relationship functioning is the mediator, we will incorporate data about their new serious partner if the participant reports one or treat that variable as missing if they report no serious partner at that follow-up.

## Results

This efficacy trial is ongoing. As of October 11, 2019, 140 dyads (individual N=280) had completed all baseline assessment components and had been randomized to either 2GETHER USA or public health practice. At the conclusion of the study, we will have enrolled and randomized 200 dyads, or 400 individuals. The final sample will be diverse in terms of race/ethnicity, HIV status, geographic region, and urban or rural location.

## Discussion

Although YMSM are the group at highest risk for HIV in the United States [1], relatively few preventive interventions have been developed that take into account the unique developmental needs of this population [78]. Further, a large proportion of new HIV infections in MSM occur in the context of serious romantic relationships [2,3], particularly among YMSM [3]. 2GETHER’s unique approach of integrating relationship education and HIV prevention for young male couples has a strong potential to reduce HIV transmission risk among those at the highest risk.

In addition to establishing the efficacy of a novel HIV prevention program for YMSM, this evaluation of 2GETHER makes several innovative contributions to HIV prevention. First, although the number of available HIV prevention programs for YMSM is on the rise [78], very few couples-based preventive interventions are available for YMSM in serious relationships. 2GETHER is also unique in that it integrates primary and secondary HIV prevention by enrolling both HIV-positive and HIV-negative individuals (in any arrangement of HIV statuses within dyads). Further, it moves beyond simply advocating for condom use by integrating information about both behavioral and biomedical prevention strategies that are relevant to individuals of any HIV status. This comprehensive approach to sexual health is especially important when working with couples because they are simultaneously trying to build dyadic intimacy and pleasure while also preventing HIV/STI transmission, goals which may be at odds with one another if they are not navigated effectively.

Even among the minimal number of available, couples-based approaches to HIV prevention for MSM, 2GETHER was the first published pilot trial of an intervention that placed an equal emphasis on relationship education and sexual health (including HIV prevention) in young male couples, and we now aim to assess its efficacy. It is our belief that if a couple can optimize their relationship functioning first (eg, improve communication and satisfaction), then they will be better able to navigate complex conversations about sexual health and safety. Indeed, we assert that our approach of leading with relationship education has the potential to provide health benefits beyond HIV prevention, including couples-based mental health treatment and substance use reduction. This is important because YMSM report fatigue associated with HIV prevention messaging, but they express a desire for programs that address their health more broadly, including relationship education [32]. Since the completion of our original pilot trial, 2GETHER is now one of several interventions that addresses both HIV prevention and relationship skills [26,79]. For example, the We Prevent program [79], which is in its initial phases of testing, is adapting relationship skills for adolescent MSM in romantic relationships in order to prevent HIV transmission.

Finally, the online adapted version of 2GETHER described in this manuscript offers specific innovation above that provided by the original, in-person version of the program. Specifically, the delivery of 2GETHER to couples across the country via videoconference provides much-needed relationship education and sexual health services for couples that often lack LGBTQ-affirming health care (eg, rural YMSM). While asynchronous telehealth programs also have the potential to increase the reach of affirming and effective services to nonurban populations, they do not offer the opportunity for participants to interact in vivo with a facilitator and receive in-the-moment feedback about the implementation of their skills. If efficacious, the approach used in 2GETHER has tremendous potential to fill the health care needs of YMSM who lack access to care in physical spaces in their area of residence.

There are several limitations inherent in the trial design. First, participants are likely not blinded to their intervention condition, given that the control condition is not attention-matched and is a single-session protocol based on existing public health practice. Second, intervention facilitators conduct sessions for both the active and control conditions. Supervision focuses on minimizing drift in condition content, but facilitators may periodically compromise fidelity to a given protocol because they facilitate sessions for both conditions. Finally, while recruiting participants from across the United States increases the ability to make inferences about generalizability, the final sample will not be representative. Despite these limitations, 2GETHER is a highly innovative and promising approach for improving relationship functioning and reducing HIV risk in young male couples. This RCT will provide important information about the efficacy of couples-based HIV prevention and relationship education, adapted for remote administration via videoconference, for a diverse group of young male couples across the United States.

## Abbreviations

LGBTQ: lesbian, gay, bisexual, transgender, and queer
MSM: men who have sex with men
PrEP: preexposure prophylaxis
RCT: randomized controlled trial
STI: sexually transmitted infection
YMSM: young men who have sex with men
